# Utility of Patch Testing and Lymphocyte Transformation Testing in the Evaluation of Metal Allergy in Patients with Orthopedic Implants

**DOI:** 10.7759/cureus.5761

**Published:** 2019-09-25

**Authors:** Logan J Richards, Alexandra Streifel, Jonathan M Rodrigues

**Affiliations:** 1 Miscellaneous, University of North Dakota School of Medicine and Health Sciences, Bismarck, USA; 2 Internal Medicine, University of North Dakota School of Medicine and Health Sciences, Bismarck, USA

**Keywords:** patch testing, metal allergy, implant, total knee arthroplasty, total hip arthroplasty, metal hypersensitivity, lymphocyte transformation test, implant failure, nuss procedure

## Abstract

Total joint arthroplasties are increasingly common orthopedic procedures performed throughout the United States. Implant failure after these procedures occurs due to a number of causes such as infection or mechanical problems, with metal hypersensitivity being an area of growing interest. The nature and mechanism of a causative relationship between metal hypersensitivity and implant failure have been unclear as it is not known whether implant failure occurs due to a previous metal allergy or metal allergy results from secondary sensitization via metal exposure in existing failing implants. Overall, there appears to be growing support and evidence for metal-hypersensitive patients having worse outcomes with regard to total hip and knee arthroplasties. However, there are conflicting recommendations (outside of Nuss procedures) for pre-implant testing for metal hypersensitivity as testing has not consistently been shown to change patient outcomes.

## Introduction and background

It has been estimated that over one million joint replacements are performed in the United States each year with joint replacement surgery accounting for the single largest category of Medicare expense [1]. With an increasingly aging population and increased worldwide life expectancy, there is an increase in the overall prevalence of musculoskeletal disease and the need for total joint arthroplasties [2]. Over 600,000 total knee arthroplasties (TKA) and over 400,000 total hip arthroplasties (THA) are performed annually in the United States alone [3,4]. TKAs comprised 56% of all joint replacement procedures, with three out of five being in females. The revision rates for TKAs have remained constant at about 8% to 10%. Approximately 465,000-512,000 hip replacements were performed in 2010 and 2011, more than double of those performed since 1992 (with around 63% occurring in females) [2]. Rates of revision surgery for THAs have remained around 20% [2]. Some of the most frequent causes of orthopedic implant failure include infection, implant size factors (mechanical issues), and placement issues of the implant. The most common cause of failure, in general, is aseptic loosening (this is followed by infection as the second most common cause) [1]. Approximately 40% of revision procedures for TKAs were due to aseptic loosening [1,5]. Aseptic loosening was also the most common cause of revision surgery in THA [1,6]. Metal hypersensitivity is thought to be a possible cause of implant failure wherein the body develops an immune response to the metal in the implant [7].

Metal hypersensitivity (allergy), as well as the association between hypersensitivity and implant failure, has long been reported, but it is a process that is still poorly understood and managed [8]. Metal-related dermatitis was first reported in 1966 with an increasing number of cases being published later [9]. In 1974, Evans et al. reported that patients can be sensitized to metal ions released by cobalt-chrome joint surfaces into the local joint tissue, which are presented to all tissues via the bloodstream, and such sensitization can be detected by patch testing (PT) [10]. Patient presentation of metal-related dermatitis may vary widely. Cutaneous hypersensitivity reactions to metal implants are most often eczematous; however, cases of vasculitis and urticaria have been documented [11-13]. These reactions may be generalized or localized. Of note, the prevalence of metal allergy in patients who have undergone THA has been found to be much higher than the general population. Specifically, it has been reported to be as high as 25% in those with well-functioning implants and 60% in those with a poorly functioning implant or implant failure [13]. The potential for a causative relationship between metal allergy and implant failure (or implant failure resulting in allergy) has been historically unclear [11].

The purpose of this paper was to review the current literature on the utility of PT and lymphocyte transformation testing (LTT) in the evaluation of metal allergy in patients with orthopedic implants pre-implantation and post-implantation. We focus on THA and TKA with a brief review of Nuss implants.

## Review

Implant metals

The most common contact allergy in the United States is metal allergy, specifically nickel. It is thought that the use of jewelry is the dominant cause of exposure [14]. Common metal alloys used in orthopedic implants are stainless steel (which contains nickel), titanium, and oxidized zirconium. Stainless steel has been used in surgical practices since the early 20th century. Type 316L stainless steel is commonly used and selected for surgical implants. It is a metal alloy with 17% to 19% chromium and 14% nickel, which allows the metal to become corrosion resistant [15]. However, its components may vary by the manufacturer [16]. Molybdenum is also added to form a protective layer against acid exposure [15]. Titanium is relatively newer in surgical practice, with its most significant advantage being its equal strength to steel while being about 50% lighter [15]. Nitinol, an alloy with 55% nickel and 45% titanium which releases nickel once implanted, is a highly malleable alloy used frequently in endovascular, cardiac and gynecological implants, but not used in orthopedic implants [11,16]. Vanadium may be included in stainless steel or titanium alloys and has an allergenic potential [16].

Metal hypersensitivity in total hip and knee arthroplasty

Presentation of metal allergy in patients with THA or TKA is usually joint swelling and pain, with overlying dermatitis at the implant site [17]. However, cutaneous reactions are not common in patients who have undergone THA. It should be noted that metal implant allergy is a diagnosis of exclusion and that at the single patient level, the presence of metal sensitivity is not necessarily indicative of future implant failure [18].

It has been found that the average lifespan of a THA was reduced from about 120 months to approximately 78 months in patients with a history of positive metal allergy testing [19]. Furthermore, although complications in metal hypersensitive patients are apparently rare, using implants that contain metals to which a patient is sensitive has been thought to trigger the events that bring about implant failure and thus shorten the lifespan of the prosthesis [20-21]. Frigerio additionally found that metal sensitization can rise as high as 6.5% after THA and TKA [20].

Granchi et. al found that PT was not able to discriminate between stable or failed THA [19]. However, they found that having a sensitivity to one or more metals, in addition to a history of delayed-type hypersensitivity, has an overall negative influence on the outcome of the implant specifically, THA failure occurs earlier. Positive PT resulted in shorter hip implant lifespan. In 2012, the same group summarized that there is a significantly higher prevalence of metal sensitization in patients with metal-on-metal total joint arthroplasty, and clinical outcomes of patients undergoing the surgery may be influenced by their hypersensitivity to the components [18].

However, not all current literature agrees with the premise that metal-hypersensitive patients have worse outcomes. In 2009, a case-control study found that the risk of surgical revision for THA was not increased in patch-tested dermatitis patients with metal allergies in comparison to those not tested [22]. It was also found that the prevalence of metal allergy was no higher in patch-tested dermatitis patients who had a THA compared to patch-tested dermatitis patients who had no procedure. Despite having a small sample size, among other limitations, they concluded that the risk of complications in metal allergy patients was limited.

Thus, there is a lack of consensus on the relationship between metal implant failure and metal allergy. However, there appears to be overall growing support and evidence for metal-hypersensitive patients having worse outcomes with regard to THA and TKA, and thus proper management will be the key. Thus, it is not known whether an implant failure occurs due to a previous metal allergy, or metal allergy results from secondary sensitization due to the release of metal components in the existing failing implants [8].

Metal hypersensitivity in the Nuss procedure

Nuss bar implants prove to be an interesting contrast to total joint arthroplasty when discussing implant metal allergy testing as Nuss bars uniquely have allergy testing recommendations based on prior studies [17,23]. Like other metal implants, metal allergy in Nuss bar patients can present variably, with some of the more common signs being dermatitis and wound infection. Implant failure in Nuss patients is much less likely due to the short amount of time they are left implanted (minimum of two years) [23]. It has been estimated that metal allergy in Nuss patients may have an incidence of about 2.2%, with some evidence of this being up to 6.4% [14,23-24]. A study examining the metal disc offered by the Nuss bar manufacturer for preoperative metal allergy evaluation of pectus excavatum patients found that it was not adequately sensitive in preoperative screening (specifically looking for nickel allergy). However, it was found that PT using metal in petrolatum was a more sensitive and accurate means of preoperative nickel allergy testing [24]. Due to the morbidity of allergic reactions in patients undergoing Nuss procedure as well as a means of potentially avoiding revision surgery, it has been suggested that PT be used on all patients prior to the Nuss procedure [23-24]. Moreover, it has also been suggested that if metal allergy testing proves to be positive to metals such as nickel, a titanium Nuss bar should be used instead [14].

Pathogenesis of metal hypersensitivity and testing modalities

The pathogenesis of metal hypersensitivity reactions is complex and involves predominately type 4 delayed-type hypersensitivity, innate immune responses, cytotoxicity, apoptosis and local lymphocyte proliferation [16]. In cases of revised TKAs, wherein the cause of implant failure was not infection, misalignment, or malposition, the surrounding tissue was shown to have high levels of T-lymphocytes [25].

Patch testing (PT) is the most readily available testing modality for metal hypersensitivity and as such is regarded as the gold standard test [26-27]. PT detects metal hypersensitivity by placing patches that contain a specific allergen on the skin and observing for the development of dermatitis following placement and removal of the patch at 48 hours and then after 72-96 hours or more, causing a type 4 reaction to allergens on the skin [16]. It is relatively easy to use and has a low cost, making it the testing modality of choice for contact dermatitis.

Lymphocyte transformation testing (LTT) works to detect metal hypersensitivity by the measurement of lymphocytes in peripheral blood that are produced in the span of 7 days following allergen exposure. The ratio of lymphocyte proliferation after allergen challenge to proliferation without allergen is expressed as a stimulation index [16,26]. Some disadvantages to LTT include high cost, limited availability, the limited number of allergens available for testing, lack of standardization, inter-laboratory variability, false-negative results in case of processing delay, and difficulty maintaining an appropriate sample for determination of lymphocyte proliferation [16,26]. It may provide some benefit for indeterminate or negative patch test results in a patient strongly suspected of having metal hypersensitivity.

The use of manufacturer-provided metal disc testing is limited by irritant, false negative and false positive reactions and is generally not recommended for clinical use [16,18].

Evaluation of metal hypersensitivity: pre-implantation

With the exception of the Nuss bar procedure, there are no current recommendations for mandatory pre-implant testing for metal hypersensitivity [14,16-17]. This is due to the fact that pre-implant testing has not shown to consistently detect patients that may have a hypersensitivity reaction following joint replacement. However, a significant portion of patients with joint implant failure test positive for metal hypersensitivity on PT or LTT [28]. This finding is complicated by the fact that some patients may develop metal hypersensitivity following placement of an artificial joint, which would not have been detected on pre-implant testing. While utilizing titanium or other similar hypoallergenic alloys for arthroplasty is an option advocated by some practice groups, titanium hypersensitivity has been reported post-implantation with symptoms including impaired fracture healing, local eczema, pain, swelling, systemic dermatitis, implant loosening, and failure, all of which have been reported to resolve with implant removal and replacement with a non-titanium implant [29-32].

A 2016 cohort study showed that, compared to matched controls with negative PT, patients undergoing TKA with positive pre-implant PT had similar rates of joint revision and reoperation [33]. This demonstrates that, even with evidence of metal hypersensitivity, there may not be an increased risk of joint rejection, revision, or reoperation. Patient screening questionnaires focusing on patient-reported history of metal hypersensitivity or questioning about reactions to jewelry and metal buttons have been found to have a positive predictive value of only around 60% on PT [34-35]. For these reasons and more, some experts suggest that broad use of pre-implantation testing based on patient-reported history of metal sensitivity and the use of patch test results to guide preoperative implant selection lacks clinical evidence and may not be feasible from a logistical and cost-efficiency standpoint [36].

Despite the lack of consensus regarding the utility of pre-implant testing, PT in patients with a demonstrated history of metal hypersensitivity prior to arthroplasty is advocated by some groups. Schalock and Thyssen surveyed members of the European Society of Contact Dermatitis (ESCD) and the American Contact Dermatitis Society (ACDS), who were predominately dermatologists, about their preferences for PT in patients with a history of metal hypersensitivity. Fifty-four percent of responders stated that they would patch test a patient with a history of metal hypersensitivity prior to implant, 38% would recommend a titanium implant for these patients and would forego testing, and 8% did not think pre-implant testing was necessary for this patient population [27]. Their results also demonstrated a difference between European and American members, with ESCD members responding more often (12%) that they would not perform pre-implant PT as compared to ACDS members (4%). In a survey of ACDS members, 88% reported rarely ever using LTT and 83% favored PT in the evaluation of patients with metal hypersensitivity reactions [16]. In one survey of orthopedic surgeons, only 11% reported routinely screening for metal hypersensitivity, 50% reported rarely referring patients for PT and the remainder stating never considering PT [37]. However, most respondents were likely to choose a different implant in patients who had a positive patch test. Another study showed consensus among orthopedic surgeons that PT is not necessary even if metal allergy is suspected with most proceeding with cobalt-chromium or stainless steel implants in patients with suspected metal hypersensitivity irrespective of PT results [38].

Nickel is the most common metal found to be positive on PT in patients with failed implants and in the general patch-tested population [39]. However, it should be noted that, when evaluating a patient with a cutaneous reaction following arthroplasty, hypersensitivity to other materials used in surgery, including bone cement components such as acrylates, benzoylperoxide, hydroquinone and N,N-dimethyl-p-toluidine; suture materials, implanted and topical antibiotics, should also be investigated and past reactions to these agents should be ascertained prior to surgery [1,16,27].

Evaluation of metal hypersensitivity: post-implantation

There are some defined criteria proposed by an international group of PT physicians for diagnosing metal hypersensitivity reactions post-implantation [16,40]. Major criteria include cutaneous eruption overlying the metal implant, chronic dermatitis that occurs weeks to months post-implantation, complete recovery after removal of the offending implant and positive patch test results to a metal used in the implant. Minor criteria include treatment-resistant dermatitis, systemic allergic dermatitis reaction, positive LTT to metals, morphology and histology consistent with allergic contact dermatitis and unexplained pain at the implant site and/or failure of the implant.

There is some debate as to whether patients with long-standing, residual pain following arthroplasty would benefit from post-implant testing [8]. While this pain may be a sign of metal hypersensitivity and indicate a reason for re-operation, there are numerous possible causes of pain following arthroplasty beyond metal hypersensitivity. The data on the relation of metal hypersensitivity to post-implant outcome and complications is conflicting. Some studies and case reports have demonstrated that even in patients with patch test verified contact allergy to nickel, the implantation of a nickel-containing arthroplasty device has caused no significant complications on long -term follow up [41,42]. Furthermore, it has also been shown that there is no association between pseudotumor formation, high serum metal-ion levels and metal hypersensitivity in patients undergoing THA [43]. However, one case series showed that up to 5% of patients developed metal-related cutaneous complications post-implant, especially in those with chromium sensitivity [44]. In another small study of 16 patients who required revision surgery due to pain, osteolysis, dislocation and/or loosening, 81 percent had metal sensitivity as determined by PT or LTT. A study based on the Danish Knee Arthroplasty Register showed that the prevalence of cobalt and chromium allergy was markedly higher in patients who had 2 or more episodes of revision surgery [45]. As previously mentioned, shorter lifespans of implants were seen in patients with positive patch reactions to metals and a history of metal hypersensitivity, with none of the patients with positive tests for bone cement components having a stable implant at the 10-year endpoint [19].

Post-implantation testing for metal hypersensitivity is recommended for patients with a history of chronic complications following arthroplasty or signs and symptoms of metal hypersensitivity that persist despite medical therapy [17]. An important factor to consider during testing post-implant patients is prosthesis induced sensitization [20]. It has been recommend that those with cutaneous reactions months following arthroplasty be patch-tested, but that those with functioning prostheses remain in place, regardless of patch test results [11]. Using the Schalock and Thyssen criteria to appropriately identify metal hypersensitivity reactions in post-implantation patients and combining PT, LTT results and potentially histopathology to identify patients who may benefit from device replacement may be a pragmatic approach [16,40,46].

Choosing the right test

Given the more widespread availability, the ability to test a variety of potential allergens, and low cost, PT is currently the most widely accepted testing modality for detection of metal hypersensitivity. However, given that PT works by introducing antigens into the dermis to induce a cutaneous reaction, there is some question as to whether PT is an accurate way to mimic the environment of the orthopedic implant [36]. In one study, 52 patients with metal hypersensitivity (diagnosed by history and patch testing) and 48 patients without metal hypersensitivity were patch tested with stainless steel and cobalt-chromium-molybdenum antigens [47]. Of the 52 patients with metal hypersensitivity, none reacted to the stainless-steel patch test and 5 reacted to the cobalt-chromium-molybdenum test. Unfortunately, this study did not utilize other forms of testing for metal hypersensitivity to ascertain if modalities besides PT had higher detection rates in this population of patients with demonstrated metal hypersensitivity.

Due to the differences between the cutaneous reactions observed in PT and the peri-implant environment, some have proposed that modalities that detect systemic hypersensitivity, such as LTT, maybe a more accurate way to test for metal hypersensitivity in orthopedics. A study by Stander et. al. investigated PT versus LTT testing in 50 individuals with a history of nickel hypersensitivity and 50 non-allergic controls [48]. With PT, 18 of the 50 nickel allergic patients tested positive, with 2 of the 50 controls testing positive as well. Of the 50 allergic subjects, 26 had positive LTT, with 2 of 50 controls testing positive on LTT. This shows that, while both testing modalities had the same rate of false positives, LTT possibly has a higher sensitivity than PT. While the subjects in this study did not undergo arthroplasty, this study suggests that LTT is possibly a better test to screen cases of metal hypersensitivity. Additionally, since LTT detects metal hypersensitivity based on lymphocyte count in peripheral blood, it is logical that this method of testing may be better suited for hypersensitivity induced by the erosion and breakdown of artificial joints. One could conclude that LTT may be a better option at detecting systemic allergies, while PT may be better at detecting dermal hypersensitivity [49]. LTT may also be a good option in cases of indeterminate hypersensitivity or in patients with joint failure of an unknown cause since it has higher sensitivity than PT. However, this may be limited by the fact that LTT requires much more time, materials, and expense and is less readily available than patch testing. While LTT and PT are complementary, they are not equivalent; patients may show metal sensitivity on one testing modality, but not the other [49].

Despite a lack of consensus regarding the utility of pre-implant testing, PT has been recommended by some groups (such as the ESCD) for patients with a demonstrated history of metal hypersensitivity prior to arthroplasty [50]. There is also an increased interest among patients and the legal system regarding the possibility of metal hypersensitivity as a cause of joint implant failure with an increased number of malpractice allegations pertaining to inadequate preoperative allergy assessment and a large number of Google hits for “metal allergy malpractice” [16]. In patients with a strong history of metal allergy, it is reasonable to consider testing, especially from a humanistic and medico-legal perspective. However, allergy testing should not take precedence over emergent scenarios or procedures that would reduce morbidity. In such situations, measures should be taken to avoid the potential allergen in the implant and the procedure should proceed without testing [16,26-27]. While the patch testing physician can help identify allergenic sensitization via testing, the decision regarding the best device that would provide the best outcome for the patient remains the expertise of the orthopedic surgeon [26].

**Figure 1:**
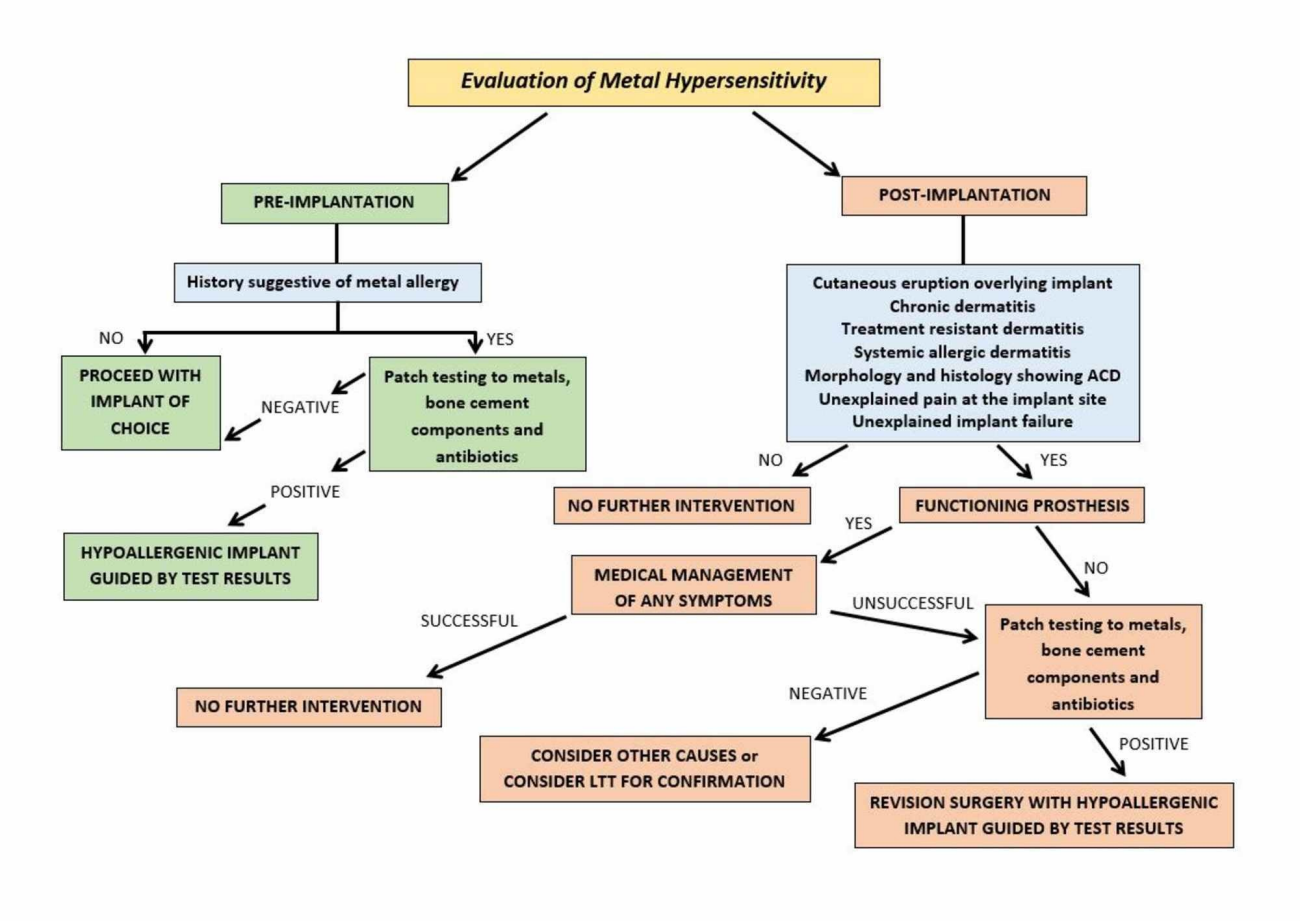
Suggested workflow for the evaluation of metal hypersensitivity in patients pre- and post-implantation with orthopedic implants ACD, allergic contact dermatitis; LTT, lymphocyte transformation test

Figure 1 shows a suggested workflow for the evaluation of metal hypersensitivity in patients pre- and post-implantation with orthopedic implants.

## Conclusions

In summary, outside of the Nuss bar procedures (with PT being recommended on all patients prior to operating), there are currently no recommendations for mandated pre-implant testing for metal hypersensitivity for procedures including total joint arthroplasties. Pre-implant testing has not yet been shown to consistently detect patients that may have hypersensitivity to metal following total joint arthroplasty. Moreover, it has not been fully elucidated as to whether an implant failure occurs due to a previous metal allergy or metal allergy results from secondary sensitization to metal components released in the existing failing implants. PT is regarded as the gold standard due to its wide availability, although it has been scrutinized for its ability (or lack thereof) to mimic the actual environment of the orthopedic implant. Although LTT has been shown to have a greater sensitivity, limitations with regard to its availability and difficulty in maintaining samples perhaps make this test more useful within the bounds of indeterminate patch test results. Post-implantation testing has been recommended for patients with signs and symptoms of metal hypersensitivity following total joint arthroplasty that persist despite medical therapy, as well as in patients with a history of chronic complications following the procedure. 
